# Screen more or screen more often? Using mathematical models to inform syphilis control strategies

**DOI:** 10.1186/1471-2458-13-606

**Published:** 2013-06-24

**Authors:** Ashleigh R Tuite, David N Fisman, Sharmistha Mishra

**Affiliations:** 1 Dalla Lana School of Public Health, University of Toronto, Toronto, Canada; 2 Institute of Health Policy, Management and Evaluation, University of Toronto, Toronto, Canada; 3 Department of Medicine, University of Toronto, Toronto, Canada; 4 Division of Infectious Diseases, St. Michael’s Hospital, University of Toronto, Rm. 4-179, 30 Bond Street, Toronto M5B 1W8, Canada; 5 Department of Infectious Disease Epidemiology, School of Public Health, Imperial College, London, UK

**Keywords:** Syphilis, Mathematical Modeling, Men Who Have Sex With Men, Sexually Transmitted Infection

## Abstract

**Background:**

Syphilis incidence among men who have sex with men (MSM) continues to rise despite attempts to increase screening and treatment uptake. We examined the marginal effect of increased frequency versus increased coverage of screening on syphilis incidence in Toronto, Canada.

**Methods:**

We developed an agent-based, network model of syphilis transmission, representing a core population of 2,000 high-risk MSM. Epidemiological and biological parameters were drawn from regional surveillance data and literature-derived estimates. The pre-intervention period of the model was calibrated using surveillance data to identify 1000 credible simulations per strategy. Evaluated strategies included: annual syphilis screening at baseline coverage, increased screening frequency at baseline coverage, and increased coverage of annual screening. Intervention impact was measured as annual prevalence of detected infectious cases and syphilis incidence per year over 10 years.

**Results:**

Of the strategies evaluated, increasing the frequency of syphilis screening to every three months was most effective in reducing reported and incident syphilis infections. Increasing the fraction of individuals tested, without increasing test frequency, resulted a smaller decline in incidence, because reductions in infectious syphilis via treatment were counterbalanced by increased incident syphilis among individuals with prior latent syphilis. For an equivalent number of additional tests performed annually, increased test frequency was consistently more effective than improved coverage.

**Conclusions:**

Strategies that focus on higher frequency of testing in smaller fractions of the population were more effective in reducing syphilis incidence in a simulated MSM population. The findings highlight how treatment-induced loss of immunity can create unexpected results in screening-based control strategies.

## Background

Urban centres in high-income countries have witnessed a re-emergence of syphilis in recent years, with the epidemic concentrated among men who have sex with men (MSM) and HIV-infected individuals [1-3]. Although the reason for this re-emergence remains unclear, proposed explanations include: increased survival of HIV-infected individuals [4,5] and changes in sexual behaviour (such as serosorting [6] or unprotected oral sex [7,8]). Despite educational campaigns promoting safer sex and serological screening, the rate of diagnosed infectious cases in many North American cities continues to increase [9-13]. Between 2000 and 2010, there was a greater than 20-fold increase in infectious (primary, secondary, and early latent) syphilis cases in the Canadian city of Toronto, with annual rates increasing from 1.9 to 38.3 cases per 100,000 males [14]. Individuals who acquire syphilis may be asymptomatic, but untreated infection can lead to ocular, auditory, and neurological complications, even during the early stages of infection [15,16]. Infectious syphilis has also been associated with acquisition and transmission of HIV [17,18].

Challenges that make syphilis difficult to control include: the development of vague or no symptoms during early infection, such that infected individuals may not seek treatment [19]; low serologic test sensitivity during the first 4–6 weeks of infection [20]; and the failure to develop protective immunity following successful treatment of infectious syphilis, such that in the presence of ongoing high-risk behaviours, individuals are at risk of syphilis re-infection [13,21-23]. In the face of these challenges, frequent syphilis screening in at-risk individuals remains the best available tool for syphilis control, by reducing the length of time an individual with syphilis remains infectious and untreated.

Clinical studies have shown that the adoption of more frequent syphilis screening among high-risk groups is feasible and increases the detection of asymptomatic infectious syphilis [19,24,25]. A recent mathematical modeling study suggested that more frequent testing may be more effective at reducing syphilis incidence than expanding the proportion of the population receiving annual screening [26]. However, these conclusions were based on a small number of model realizations of an epidemic in a large MSM population [26]. Further evaluation is required to determine whether the findings [26] are robust to parameter uncertainty (using a larger number of simulations), model uncertainty (using a different model structure), and are applicable to a smaller, core population of high-risk MSM at greatest risk of re-infection.

Given the burden of syphilis in MSM and the clear need for more impactful interventions to reduce incidence and achieve long-term epidemic control, we created a mathematical model of syphilis transmission dynamics in Toronto, Canada to estimate the potential effectiveness of different syphilis screening strategies. Specifically, we evaluated whether expanding population coverage or increasing test frequency among individuals already undergoing routine syphilis screening would be more effective in a population of high-risk MSM.

## Methods

### Transmission model overview

We modeled the dynamics of syphilis in MSM in Toronto, Canada, using an agent-based approach. We modeled Toronto because it is one of the cities most affected by the recent syphilis resurgence in the MSM population [14], and sexual behaviour data are readily available for high-risk MSM [27]. Of the approximately 500 men diagnosed with infectious syphilis in Toronto in 2010, 89% reported sex with men as a risk factor, and up to 48% were co-infected with HIV [14]. Data used to model the behavioural component of the model (discussed in more detail below) were derived from a second generation surveillance study that used a venue-based recruiting strategy to enrol MSM from major urban centres, such that study participants were more likely to identify with the gay community [27]. We use the term high-risk to emphasize that the modeled population likely differs from the general population of MSM in Canada with respect to frequency of unprotected anal intercourse and other high-risk behaviours, and has an incidence of HIV infection that is approximately five times higher than that observed among all MSM in this region of Canada [27,28]. An agent-based approach allowed for the explicit modeling of complex sexual networks and recording of sexual histories and health states of discrete individuals over time. The model was constructed using the Any Logic software package (http://www.xjtek.com/anylogic/).

The model represented a population of 2,000 high-risk MSM, with individuals forming a network of sexual contacts along which the transmission of syphilis occurred. Based on local HIV prevalence estimates in MSM, 20% of the population was assumed to be HIV positive [29]. Each modeled individual was described by an infection transmission component and a partnership component, as described in greater detail below. Model parameters were drawn from the literature and were Toronto-specific, wherever possible, or were based on expert opinion in the absence of data (Table 1). Since parameters were based on aggregate and publicly available estimates, the study did not require institutional ethical approval.

**Table 1:** Model parameters

**Parameter**	**Details**	**Value**	**Distribution**	**Source**
			**Type**	
**Population characteristics**				
Population size		2,000		
Time spent in model (years) (min, max, mode)		1, 34, 17	Triangular	[27]; assumption
Proportion of MSM who are HIV positive		0.2		[29]; assumption
**Syphilis natural history**				
Probability of transmission (per act) (min, max, mode)	Penile-anal/Penile-oral	0.01, 0.05, 0.014	Triangular	[26]; assumption
Incubation period (days)		21-28	Uniform discrete	[30]
Infection/infectious period (days)				[16,31,32]
	Primary	45-60	Uniform discrete	
	Secondary	100-140	Uniform discrete	
	Early latent*	365		
	Recurrent	90		
	Late Latent*	Until end of life in model		
Probability of recurrent syphilis		0.25		[33]
Duration of protective immunity following				[21,34,35]
treatment (years)				
	Primary or secondary syphilis	0		
	Latent syphilis	5		
**Partnership characteristics**				
Number of partners in past 6 months (proportion of population in each category)				[27]
	1	0.28		
	2-9	0.48		
	10-29	0.15		
	30-75	0.09		
Maximum number of partnersin past 6 months (by partner number category) (min, max, mode)				
	1	1		Assumption
	2-9	2,9,8	Triangular	
	10-29	10,29,25	Triangular	
	30-75	30,75,50	Triangular	
Duration of partnership (days) (min, max, mode)				[27]; Assumption
	Casual	1,2,1	Triangular	
	Regular	7,3000,365	Triangular	
**Behavioural characteristics**				
Frequency of anal sex (per day)				[26]
	Casual partnership	0.7		
	Regular partnership	0.3		
Frequency of oral sex (per day)				[26]
	Casual partnership	1		
	Regular partnership	0.3		
Probability of condom use (anal sex)				[2,27]
	HIV-concordant	0.5		
	HIV-discordant	0.8		
Condom efficacy	Assume condom use for anal intercourse only	0.9		[36]
**Test and Treatment characteristics**				
Probability of seeking medical care for symptoms				[19,37]
	Primary	0.25		
	Secondary	0.60		
Time to treat (days)	Primary	3-56	Uniform	[38]; assumption
	Secondary	1-57	Uniform	
Proportionof population screened routinely for				[19,24,39,40];
syphilis				assumption
	HIV positive	0.5		
	HIV negative	0.2		
Test sensitivity	Treponemal-specific screening test	0.95		[41,42]
Probability of partner notification				Assumption
	Casual partner	0.1		
	Regular partner	0.6		
Trace-back period for partner notification				[43]
(months)				
	Primary	3		
	Secondary	6		
	Early latent	12		
Time from index case identification to screening of named partner(s) (days)		3-21		Assumption

*Not infectious.

### Infection transmission component

**Figure 1:**
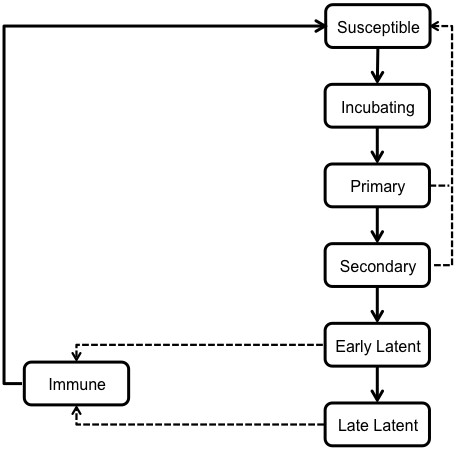
**Schematic of the infection transmission component of the model.** Each box represents a discrete health state for an individual, with transition times between health states defined in Table 1. Dashed lines represent transitions that occur following successful treatment of syphilis-infected individuals.

The infection transmission component of the model (Figure 1) represented an individual’s health state and incorporated the stages of syphilis infection: susceptible, incubating, primary, secondary, early latent, late latent, and immune. Uninfected individuals became infected via a transmission event following contact with an infected partner. Individuals progressed through the various stages of syphilis, with parameters describing the transitions between states listed in Table 1.

Men were tested for syphilis if they sought medical care for symptoms or accepted screening, with a likelihood of correct diagnosis dependent on test sensitivity [20,44]. We did not include the diagnosis of false positive cases. A proportion of regular and casual contacts of diagnosed cases were identified and screened via contact tracing, with trace back periods dependent on disease stage, as per Canadian guidelines (3 months, 6 months, and 1 year, for primary, secondary, and latent syphilis, respectively) [43]. At baseline, HIV-positive men were more likely to undergo regular screening than HIV-negative men [29,45]. Successful treatment of infectious individuals was assumed to abort the development of protective immunity for men with primary or secondary syphilis, such that they returned directly to the susceptible state following effective treatment [34]. Individuals with latent syphilis who received treatment were assumed to be immune from re-infection for 5 years [21]. We assumed that an individual could not become `super-infected’ with syphilis if he was already infected or had recently received treatment for latent infection and was transiently immune.

### Partnership component

The partnership component described an individual’s sexual network and was based on Toronto-specific data from the Lambda survey [27]. This biological and behavioural survey was conducted on a convenience sample of 2,500 gay and bisexual men in Toronto and Ottawa, who were enrolled through targeted gay venues [27]. The survey collected data on sexual and health-seeking behaviours, including condom use, known HIV status, and syphilis testing [27]. Behavioural parameters were estimated for men reporting one or more sexual partners in the past six months.

In the model, each individual was assigned a number of desired partners per six-month period, and formed partnerships with other partner-seeking individuals in the simulated population. Partnerships could be concurrent or serial. Casual and regular partnerships differed by frequency of sexual contact and partnership duration. We differentiated between casual and regular partnerships for the sake of applying contact tracing (i.e., we assumed different probabilities of identification and treatment of regular versus casual partners) and for the application of different behavioral characteristics, such as condom use and frequency of sexual contact. Condom use was assumed to be nil during oral sex [27]. We assumed a greater likelihood of condom use for partnerships involving men with discordant HIV status, compared to partnerships involving HIV concordant men [27].

### Model calibration

Each stochastic model realization represents one result out of many possible epidemic trajectories. We selected and analyzed simulated epidemics that reproduced the annual case detection rate of primary, secondary, and early latent syphilis among high-risk MSM in Toronto between 2006 and 2010. We used the reported number of cases of infectious syphilis among males in Toronto during this time period divided by the estimated size of the Toronto MSM population [28,29,46] to provide lower bound estimates of the expected burden of detected disease in the modeled population of 2000 men over a five year period (~50-75) [14]. The upper bound was based on annual number of cases of infectious syphilis diagnosed in sentinel surveillance clinics (Leo Mitterni, personal communication), which are expected to serve higher-risk MSM; using these data, we expected to see approximately 10-fold more cases in the modeled population over the five-year pre-intervention period than the number estimated using city of Toronto surveillance data (i.e., ~750 cases). This is comparable to estimates for other sexually transmitted infections (STIs) in epidemiological core groups [47]. We therefore regarded model realizations as “credible” if they produced detected case counts that fell within the uncertainty bounds defined above during the pre-intervention period (i.e. between 50–750 reported infections over the five year pre-intervention period in our population of 2000 men). A total of 1000 of these credible model realizations were used for the analysis of each intervention. During the calibration process, parameters were varied within plausible limits (as described in Table 1). Individual parameter values in the calibrated model realizations covered this same range and distribution for key input parameter values, but were used jointly in individual realizations in a manner that optimized model fit to observed data.

### Control strategies and analysis

We evaluated the impact of increasing either the coverage or frequency of syphilis screening in high-risk MSM (Table 2) by comparing the projected number of reported and incident syphilis cases under these interventions to the base case of annual screening at current coverage (Strategy A). Increasing annual population coverage by 10% (Strategy B) and increasing frequency of screening in those already being screened (Strategy C) were considered feasible, pragmatic interventions. However, because the increased frequency strategies required many more tests than the expanded coverage scenario, we also evaluated the impact of dramatically increasing population coverage for annual testing (Strategy D), such that the total number of tests performed per year was approximately equivalent to the number of test performed under the increased frequency scenarios. Note that screening 100% of the population annually resulted in 80 fewer tests per year than screening individuals already seeking screening every 3 months.

**Table 2:** Syphilis screening strategies evaluated in the model

**Intervention**	**Description**	**Details**
(A) Base case	Screen every 12 months	• 20% of HIV-negative individuals screened
		• 50% of HIV-positive individuals screened
		• 60% of regular and 10% of casual partners of infectious index cases treated
		• 520 tests performed annually
(B) Increase coverage of screening	Increase coverage by 10%	Same as (A), but:
		• 30% of HIV-negative individuals screened
		• 60% of HIV-positive individuals screened
		• 720 tests performed annually
(C) Increase frequency of screening	Screen every 6 or every 3 months	Same as (A), but
		• Frequency of screening in population is increased to every 6 (1040 tests annually) or 3 (2080 tests annually) months*

(D) Equivalent number of tests	Screen a proportion of the population every 12 months such that the total number tests performedis equivalent to (C)	To equal every 6 months:
		• 100% of HIV-positive individuals screened and 40% of HIV-negative individuals screened (1040 tests annually)
		To equal every 3 months:
		• 100% of the population screened (2000 tests annually)*

*Note that there are 80 extra tests required annually for the screen every 3 months strategy, compared to the equivalent number of tests strategy with 100% annual coverage.

All interventions were initiated in 2011, with immediate scale-up, and were sustained over a 10-year period. Intervention impact was measured as the prevalence of detected infectious (primary, secondary, and early latent) cases per year over the intervention period. We also estimated the annual incidence of syphilis. To characterize the uncertainty around these estimates, we constructed 95% bootstrap confidence intervals with 1000 replications, using sampling with replacement from the 1000 runs conducted for each model scenario.

## Results

In total, approximately 2500 simulations were required to produce 1000 “credible” epidemics for each scenario. Of the discarded simulations, 37% fell below the lower limit and 63% produced detected cases above the upper limit. None of the credible epidemics achieved local elimination over the subsequent 10 years.

**Figure 2:**
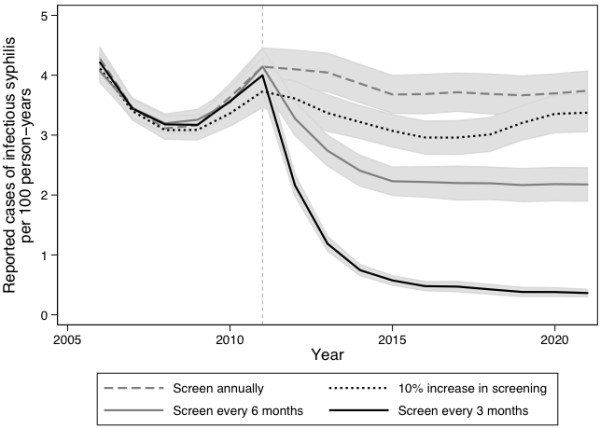
**Model-projected annual rates of reported infectious syphilis.** Results are based on 1000 realizations of each intervention scenario and are presented as mean values with corresponding 95% uncertainty bounds. Prior to 2011, all scenarios included annual screening only, with the specified interventions implemented at the start of 2011 (indicated by a dashed line).

Using model realizations that produced reported case counts within our target calibration range for the time period between 2006 and 2010, we examined the effect of increasing the frequency or coverage of screening in the model population for a 10-year period beginning in 2011. All strategies were projected to reduce the number of reported infectious syphilis cases, compared to the base case scenario of continuing to screen a fixed proportion of the population annually (Figure 2). Results were similar for incident syphilis cases (data not shown). Increasing the frequency of screening to every 3 months in men already undergoing testing was the most effective strategy for reducing infectious syphilis cases in this population; over the 10-year intervention period, 3 monthly screening was projected to avert approximately 650 incident syphilis cases relative to the base case, compared with 300 and 125 cases averted with 6 monthly or expanded annual coverage, respectively.

**Figure 3:**
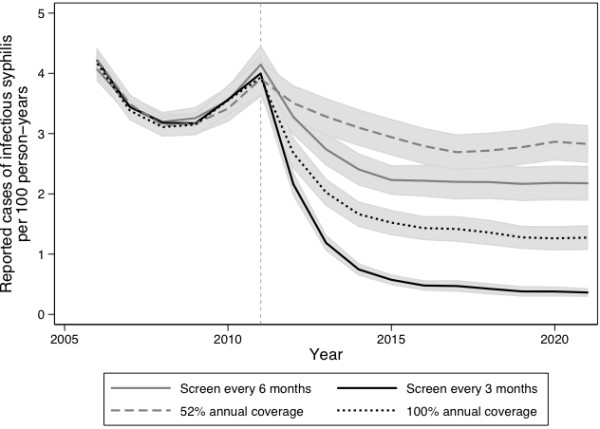
**Model-projected annual rates of reported infectious syphilis under equivalent test volume strategies.** Results are based on 1000 realizations of each intervention scenario and are presented as mean values with corresponding 95% uncertainty bounds. Prior to 2011, all scenarios included annual screening only, with the specified interventions implemented at the start of 2011 (indicated by a dashed line). 3-monthly and 100% annual screening (black lines) required approximately the same number of screening tests annually, as did the 6-monthly and 52% annual screening (grey lines).

**Figure 4:**
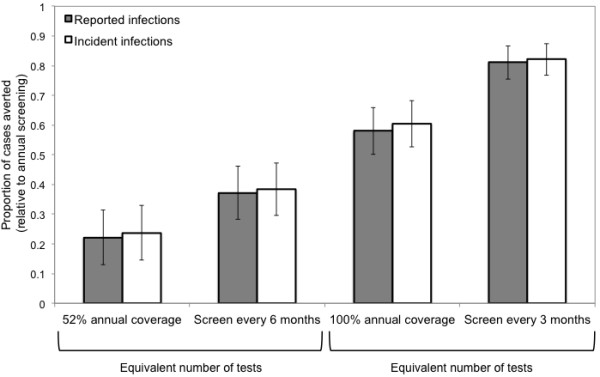
**Expected reduction in infectious syphilis cases following implementation of different intervention strategies.** The proportion of cases averted was calculated relative to the expected number of cases in the base case scenario (no increase in frequency or coverage of screening). Proportion of cases averted is presented for both diagnosed cases and incident cases (reported and unreported), and is calculated using the mean value of 1000 realizations for each intervention scenario. Error bars represent 95% uncertainty bounds. Strategies requiring the same number of annual tests are indicated.

Comparing strategies that required approximately the same annual number of tests, more frequent testing in men already accessing screening was projected to be more effective than expanding the proportion of the population that received annual testing (Figure 3). The proportions of cases averted, relative to the base case, were greater for the more frequent screening strategies than when the equivalent number of tests was applied to expanded coverage over the intervention period (Figure 4).

## Discussion

Using a dynamical model of syphilis transmission in a core group of MSM that was parameterized with the best available data on the epidemiology of the current epidemic and disease natural history, we evaluated plausible screening strategies that might be employed for epidemic control. We found that increasing test frequency in at-risk MSM who already access screening, rather than expanding outreach to provide screening to under-screened individuals, would be the optimal means of reducing syphilis incidence.

While the finding may appear counterintuitive, it is consistent both with the observed rebound in syphilis rates that occurred in Vancouver in the context of dramatic expansion of empirical treatment efforts [48], and with the hypothesis that recurrent syphilis epidemics occur as a result of emergence of large populations of susceptible, rather than infectious, individuals [35,49]. Our findings are expected under a disease natural history model that includes immunity to super-infection in individuals with late syphilis, and which allows infected individuals to become re-infected (and infectious) after treatment. Rebound in such a system occurs because creation of a large population of newly syphilis-susceptible individuals, via treatment, provides the necessary population at risk for a marked increase in new infections. By contrast, screening and treating a limited subset of the population more frequently and rapidly continually truncates infectiousness in the population, allowing case numbers to decline.

By incorporating sexual network data and capturing the indirect effects of interventions, such as the downstream prevention of cases following treatment of a single individual, we are able to evaluate the potential impact of changes in syphilis screening on disease dynamics. Previous modeling studies of syphilis transmission have provided important qualitative and quantitative insights for epidemic control by exploring the influence of key parameters on syphilis rebound [1,21,48,50]. These studies demonstrated that a rapid loss of immunity following treatment, faster turnover in high-risk groups, and higher rates of partner exchange would lead to an earlier and larger rebounds in syphilis incidence [1,48,50]. The observed transient drop in cases observed prior to initiation of the interventions (in Figures 2 and 3) likely represents damped oscillations occurring in the system, as would be expected to occur in a dynamic model with replacement of removed individuals with susceptibles.

To our knowledge, only one previous study has performed a similar examination using an agent-based model of syphilis in Australian MSM, and demonstrated the potential impact of more frequent screening in MSM, compared to increasing the proportion of MSM screened [26]. Our model differs from the Australian study, in that we focused on a core group of high-risk MSM, with higher expected rates of partner change and connectivity. Our results therefore confirm that the Australian findings are robust to parameter and model uncertainty, and are generalizable to a smaller, core population of high-risk MSM, in a different epidemic context. Our findings lend epidemiological support to current STI guidelines that recommend frequent syphilis screening in MSM [43,51].

Our model is subject to limitations, including uncertainty in model parameters. When the appropriate data were available, parameters were drawn from distributions to account for this uncertainty. Although we included results from a large number of model realizations for each strategy, we did not explicitly evaluate the impact of stochastic uncertainty on model results within each parameter set. However, the impact of stochasticity on the results appears to be small, likely due to the reasonably large number of simulations performed. The partnership component of the model was parameterized using data from a cross-sectional study, and thus may not capture changes in behaviour over time, and their consequent impact on the syphilis epidemic in Toronto’s MSM population. Although our model included HIV status, we did not attempt to evaluate the synergistic impact of syphilis testing and treatment on syphilis-HIV co-infection. An important next step is to incorporate costs and evaluate the cost-effectiveness of these strategies.

## Conclusions

In summary, a model that incorporates the best available data on syphilis transmission in MSM core groups in a large urban centre suggests that syphilis screening campaigns will be most successful if they focus on reducing the interval between tests in high-risk MSM, rather than focusing on outreach to increase the proportion of the population screened.

## Competing interests

The authors declare that they have no competing interests.

## Authors’ contributions

All authors were involved in the study conception and design, analysis and interpretation of data, and drafting of the manuscript. AT built the model and analyzed output data. SM and DF assisted with data acquisition and the critical revision of the manuscript for important intellectual content. All authors read and approved the final manuscript.

## Pre-publication history

The pre-publication history for this paper can be accessed here:

http://www.biomedcentral.com/1471-2458/13/606/prepub
